# Public trust and moral perceptions as predictors of anticipated preventive behavior toward future epidemics among adults in Riyadh, Saudi Arabia: a cross-sectional study

**DOI:** 10.3389/fpubh.2026.1950414

**Published:** 2026-10-07

**Authors:** Hammam Fahad Alkanhal, Elsir Abdelmutaal Sirelkhatim Mohammed, Ziyad Obaid Alharbi, Munirah Hassan Alalwan

**Affiliations:** 1 Saudi Board for Preventive Medicine, Riyadh Second Health Cluster, Riyadh, Saudi Arabia; 2 Riyadh Third Health Cluster, Riyadh, Saudi Arabia; 3 Infection Control and Environmental Health Administration, King Fahad Medical City, Riyadh, Saudi Arabia

**Keywords:** anticipated behavior, epidemic preparedness, future epidemics, moral perceptions, preventive behavior, public trust, Saudi Arabia

## Abstract

**Background:**

Future epidemic preparedness depends partly on public willingness to follow preventive measures. This study examined whether public trust and moral perceptions were associated with anticipated preventive behavior toward future epidemics among adults in Riyadh, Saudi Arabia.

**Methods:**

A cross-sectional survey included 256 adults. Three 10-item constructs measured public trust, moral perceptions, and anticipated behavior using five-point Likert scales. Internal consistency, correlations, subgroup comparisons, multiple regression, and a fully adjusted general linear model were evaluated. Regression assumptions were examined, and HC3 robust and influence-sensitivity analyses were conducted.

**Results:**

Mean scores were 3.903 ± 0.651 for public trust, 3.947 ± 0.630 for moral perceptions, and 4.361 ± 0.728 for anticipated behavior. Anticipated behavior correlated with public trust (*r* = 0.671, *p* < 0.001) and moral perceptions (*r* = 0.742, *p* < 0.001). The primary model explained 60.4% of anticipated-behavior variance, *F*(2,253) = 192.693, *p* < 0.001. In the full-sample adjusted model (*N* = 256), public trust (*B* = 0.357, standardized beta = 0.319, *p* < 0.001) and moral perceptions (*B* = 0.495, standardized beta = 0.428, *p* < 0.001) remained positively associated with anticipated behavior. Household income was not significant overall (*p* = 0.419). HC3 robust estimation and exclusion of observations flagged by the conservative Cook’s-distance threshold did not change the conclusions.

**Conclusion:**

Public trust and moral perceptions were independently associated with anticipated preventive behavior, with moral perceptions showing the larger adjusted association. Preparedness strategies should combine credible institutional communication with culturally appropriate messages emphasizing collective responsibility and protection of vulnerable groups.

## Introduction

Emerging infectious disease outbreaks have shown that epidemic control depends not only on surveillance, clinical capacity, testing, and pharmaceutical interventions, but also on sustained public adherence to preventive measures. Behavioral responses are shaped by risk perception, institutional credibility, leadership, social norms, and collective responsibility, making behavioral preparedness a core component of epidemic readiness (1, 2). Saudi Arabia is a particularly relevant setting because of its experience with MERS-CoV and COVID-19, centralized health communication, high digital connectivity, and the coexistence of official and informal information sources (3–6).

Public trust may support compliance when people view health authorities as competent, transparent, fair, and acting in the public interest. It can increase acceptance of changing recommendations, vaccination, isolation, and other measures, whereas perceived inconsistency, limited transparency, conflicts of interest, and misinformation may weaken cooperation (6–8). In Saudi Arabia, reliance on official information sources during COVID-19 was associated with widespread adoption of preventive practices, indicating that institutional credibility may remain an important asset in future emergencies (6).

Preventive behavior also has a moral dimension because individual actions can reduce harm to family members, vulnerable groups, and the wider community. Collective identity, prosocial norms, and responsibility toward others can therefore complement institutional trust as drivers of adherence (1, 5). Within the Theory of Planned Behavior, trust may shape attitudes toward official guidance, while moral perceptions may operate through personal and subjective norms concerning the duty to protect others (9, 24–26). This interpretation is also consistent with Saudi evidence linking perceived benefits, barriers, and cues to action with preventive practices (10).

International and regional evidence supports the relevance of these constructs. Cross-national studies have linked government and interpersonal trust with preventive and prosocial behavior, while qualitative evidence has highlighted clear rationale, community protection, and concern about harming vulnerable people (11–13). Vaccine studies similarly show that acceptance is sensitive to institutional trust, perceived safety, misinformation, and confidence in approval processes; these concerns have been especially variable across Middle Eastern populations (14–20). Together, this literature suggests that trust and moral responsibility may influence both routine non-pharmaceutical measures and more contested behaviors such as vaccination.

Saudi studies further show high reliance on official sources and broad adoption of preventive measures, while also identifying variation in knowledge, perceived benefits and barriers, anxiety, vaccine confidence, and susceptibility to misinformation (6, 8, 10, 21–23). This combination makes it important to examine trust and moral perceptions together rather than treating compliance as a simple consequence of knowledge or policy enforcement. It also recognizes that future epidemics may generate different levels of fear, fatigue, uncertainty, and skepticism from those observed during COVID-19. The continued global circulation of SARS-CoV-2 and lessons from the WHO review of the COVID-19 response in the Eastern Mediterranean Region further underscore the need for sustained preparedness for future health emergencies (25, 26).

A gap therefore remains in understanding how adults in Riyadh anticipate responding to future epidemics and whether public trust and moral perceptions independently contribute to that readiness. This study examined the association of public trust and moral perceptions with anticipated preventive behavior among adults in Riyadh, Saudi Arabia. We hypothesized that higher public trust and stronger moral perceptions of communal responsibility would each be associated with greater anticipated adherence to preventive measures.

## Materials and methods

### Study design and setting

A quantitative, observational, cross-sectional survey was conducted to examine the association of public trust and moral perceptions with anticipated behavioral responses toward future epidemics among adults in Riyadh, Saudi Arabia. Riyadh was selected as the study setting because it includes a demographically diverse urban population and serves as a national center for health services, education, media, and public health communication. Data collection was conducted during April and May 2026. The study used a non-interventional design; no clinical procedures, treatments, or experimental exposures were applied. The design was aligned with the primary objective of estimating levels of public trust, moral perceptions, and anticipated epidemic-related behavior and testing whether public trust and moral perceptions independently predicted anticipated adherence to preventive measures.

### Participants and recruitment

A non-probability convenience sampling method supported by snowball distribution was used. The target population consisted of adult residents of Riyadh. Participants were eligible if they were aged 18 years or older, were current residents of Riyadh, were able to read and understand Arabic, and provided informed consent before starting the questionnaire. Individuals younger than 18 years, non-residents of Riyadh, and those unable to complete the questionnaire because of language or communication barriers were not eligible. Recruitment was conducted through online survey distribution and community-based access routes, including social media platforms, messaging applications, and QR-code links distributed through accessible public or community settings. Snowball sharing was permitted to expand reach across age, education, income, and employment subgroups.

### Sample and data screening

The sample size was calculated using the single-proportion formula for cross-sectional studies, n = Z^2^ × p × (1 − p)/d^2^, where n represented the minimum required sample size, Z was the standard normal value at a 95% confidence level, p was the expected proportion of the main trust- or behavior-related outcome, and d was the accepted margin of error. Based on previous Saudi evidence showing that approximately 78% of participants trusted or supported government mitigation measures during COVID-19 (21), the expected proportion was set at *p* = 0.79 for the sample-size calculation after rounding. Using *Z* = 1.96 and *d* = 0.05, the calculation was *n* = (1.96)^2^ × 0.79 × 0.21/(0.05)^2^, which produced an estimated minimum sample size of approximately 255 participants.

A total of 256 completed responses were received through the electronic questionnaire, and all 256 were retained in the final analytic sample. The required core questionnaire fields were mandatory and used predefined response options; consequently, no completed submission had missing data in the 30 core Likert items. After data collection, the exported records were screened for completeness, allowable response ranges, response consistency, duplicate response profiles, and unusual response patterns. Thirty-four records showed straight-line response patterns and four pairs had identical non-timestamp response profiles. These records were reviewed but were not excluded because neither straight-line responding nor similarity between anonymous response profiles, in isolation, was considered sufficient evidence of an invalid response. No Likert response was outside the permitted 1–5 range, and no participant-level response was excluded. The final analytic sample therefore comprised 256 participants and met the calculated minimum sample-size requirement.

### Data collection instrument

Data were collected using a structured self-administered questionnaire developed and adapted for the present study. The instrument was not adopted as a complete pre-existing scale. Instead, constructs, questions, and behavioral domains identified in published Saudi and international literature were selected and reformulated to match the study objectives, the Saudi context, and the prospective framing of behavior toward future epidemics. Public-trust items were principally informed by Halma and Guetzkow (7) and Alkhaldi et al. (6); vaccine-related items by Nour and Natto (22); adherence and epidemic-perception items by Almalki et al. (23) and Alkhaldi et al. (6); and misinformation-related items by Alasmari et al. (8). The wording and organization of the final three 10-item constructs were developed specifically for this study.

The questionnaire was originally prepared in English and translated into Arabic by a professional Arabic-English translator working through a licensed professional translation office in Saudi Arabia. Back-translation was performed by a second professional translator who was different from and independent of the forward translator. The back-translated English version was compared with the original English questionnaire to assess semantic and conceptual equivalence. Wording discrepancies were reviewed and reconciled before the Arabic questionnaire was finalized. The translation service was provided through Creative Translator, a licensed professional translation office in Saudi Arabia (License No. 203005000119).

### Content validity

Content validity was evaluated independently by three subject-matter experts in preventive medicine, epidemiology, and research methodology. Each expert rated all 30 items on a four-point scale for relevance, clarity, comprehensibility, cultural suitability, and appropriateness. For relevance, ratings of 3 or 4 were classified as content-valid. Twenty-seven items achieved an item-level content validity index (I-CVI) of 1.00, and three items achieved an I-CVI of 0.67, producing a scale-level average content validity index (S-CVI/Ave) of 0.967. Mean expert ratings were 3.56 for clarity, 3.57 for comprehensibility, 3.92 for cultural suitability, and 3.67 for appropriateness. Items receiving an I-CVI of 0.67 were reviewed with particular attention to wording and the intended reverse-scored content before the instrument was finalized.

The final analytic questionnaire contained a sociodemographic section and 30 Likert-scale items distributed equally across the three main constructs. The sociodemographic section collected age in years, gender, marital status, education level, employment status, and monthly household income. The public trust scale included 10 items covering confidence in Saudi health authorities and health experts, perceived transparency and fairness of health authorities, scientific basis of health decisions, preference for official Saudi sources when social media information conflicted with official guidance, trust in national media compared with social media, willingness to follow public health instructions and encourage family members to do the same, perceived public-interest orientation of Saudi health authorities, perceived public-interest orientation of the World Health Organization, caution or questioning of motives behind some health recommendations, and perceived acceptability of not following policy if it clearly contradicted Islamic rules.

The moral perceptions scale included 10 items assessing personal responsibility for protecting others during health emergencies, viewing recommended vaccines as a responsible action to protect the community, acceptance of minor vaccine side effects to protect higher-risk groups, the role of religious beliefs when guidance is supported by trusted religious authorities, Saudi cultural values supporting health interventions that protect others, perceptions that some preventive measures may exceed what is really needed, the possibility of not following every health measure when perceived risk is very low, moral acceptability of ignoring official health guidance in some situations, moral unacceptability of spreading unverified health information during epidemics, and the belief that people should follow health recommendations when they are linked to protecting others.

The anticipated behavior scale included 10 items measuring willingness to follow preventive health measures and authority instructions in future epidemics, maintaining hand hygiene and cleaning frequently touched surfaces, avoiding crowded places and unnecessary travel or non-essential social visits during outbreaks, practicing social distancing and wearing a face mask when recommended, willingness to receive recommended vaccines, ability and willingness to self-isolate if infected, use of official Saudi digital health platforms during future epidemics, prompt symptom reporting or testing when infection is suspected, avoiding sharing unofficial health information through social media, and the reverse-coded belief that future epidemics may not worsen even if some people do not fully follow guidance.

### Measures and scoring

All construct items were measured using a five-point Likert response format ranging from 1 = strongly disagree to 5 = strongly agree. Higher scores represented higher public trust, stronger responsibility-oriented moral perceptions, and stronger anticipated adherence to preventive behaviors after reverse coding negatively worded or skepticism-oriented items. The reverse-coded items were T9 and T10 in the public trust scale, M6 to M8 in the moral perceptions scale, and B10 in the anticipated behavior scale. Mean scores were computed for each construct by averaging valid item scores after reverse coding. An overall total score was computed by averaging the 30 construct items. For descriptive classification, mean scores were categorized as low (1.00–2.33), moderate (2.34–3.67), or high (3.68–5.00).

### Reliability and data quality

Internal consistency reliability was assessed using Cronbach’s alpha for each main scale and for the total 30-item scale after applying the relevant reverse coding. The reliability analysis showed acceptable to excellent internal consistency in the final analytic sample: alpha = 0.773 for public trust, alpha = 0.706 for moral perceptions, alpha = 0.886 for anticipated behavior, and alpha = 0.912 for the total scale. Item-total diagnostics were also examined to evaluate the contribution of individual items to the internal consistency of each construct.

### Statistical analysis

Data were analyzed using IBM SPSS Statistics version 30. Frequencies and percentages summarized categorical variables, while means, standard deviations, medians, interquartile ranges, and ranges summarized continuous variables and scale scores. Because formal distribution tests were significant for the main scores, both Pearson and Spearman correlations were retained. Group differences were assessed using independent-samples t-tests and one-way analysis of variance, and chi-square tests examined associations between categorical level classifications. Multiple linear regression estimated the associations of public trust and moral perceptions with anticipated behavior. Regression diagnostics included tolerance, variance inflation factors (VIF), the Durbin-Watson statistic, residual-versus-fitted and Q-Q plots, the Breusch-Pagan test, and Cook’s distance. Because heteroscedasticity was detected, HC3 heteroscedasticity-robust standard errors were additionally estimated. Observations exceeding the conservative Cook’s-distance screening threshold of 4/N were examined in a sensitivity analysis rather than deleted automatically.

The fully adjusted general linear model included age, gender, marital status, education, employment status, and household income. Participants selecting “prefer not to answer” for household income had complete questionnaire and outcome data but no disclosed income value. The original complete-case specification treated this response as unavailable income information (*N* = 208). The full-sample specification retained “prefer not to answer” as a separate income-response category (*N* = 256), thereby adjusting for the pattern of non-disclosure while preserving the full analytic sample. The full-sample specification was used for the revised primary adjusted results, and the complete-case specification was retained as a sensitivity analysis. Statistical significance was set at *p* < 0.05 using two-sided tests.

### Ethical considerations

The study followed the principles of voluntary participation, anonymity, confidentiality, and minimal risk. The first page of the questionnaire provided information about the study purpose, eligibility criteria, voluntary participation, expected risks and benefits, confidentiality, and the right to discontinue without penalty. Consent was obtained through an implied consent process; participants indicated agreement by proceeding to the anonymous questionnaire, and no signatures were collected because no direct identifiers were obtained. Ethical approval was obtained from the Institutional Review Board of King Fahad Medical City - Riyadh Second Health Cluster (IRB Log No. 25-565C; approval date: November 2, 2025; category of approval: expedited).

No personal identifiers were collected in the survey dataset. Responses were stored in password-protected electronic files accessible only to the research team. Data were used only for research purposes and were planned for secure retention for no more than 5 years before deletion. Participants were informed that they could skip any question they did not wish to answer and could stop participation at any point without consequence.

## Results

### Sample characteristics and data quality

The study received 256 completed survey responses, all of which were retained after quality screening. No participant was excluded for ineligibility, questionnaire incompleteness, duplication, or another data-quality reason. All 256 respondents had complete valid data for the 30 core Likert items. The mean participant age was 35.69 ± 10.10 years (range, 18–72 years); 154 (60.2%) were male and 102 (39.8%) were female. Most participants were married (58.6%) and held a bachelor’s degree (55.1%), and the largest occupational category was healthcare workers (21.5%). More than one third reported monthly household income above 15,000 SAR (36.3%), while 48 participants (18.8%) selected “prefer not to answer” (Table 1).

**Table 1:** Condensed analytic sample and participant characteristics (*N* = 256).

Characteristic	Category/statistic	n or value	%/range
Age, years	Mean ± SD	35.69 ± 10.10	Range: 18–72
Gender	Male	154	60.2%
	Female	102	39.8%
Marital status	Single/unmarried	95	37.1%
	Married	150	58.6%
	Divorced/widowed	11	4.3%
Education	Less than high school or high school diploma	52	20.3%
	Bachelor’s degree	141	55.1%
	Master’s or doctoral degree	63	24.6%
Employment status	Government employee (civilian)	46	18.0%
	Healthcare worker	55	21.5%
	Private sector or education sector	51	19.9%
	Student	32	12.5%
	Other employment categories	72	28.1%
Monthly household income	More than 15,000 SAR	93	36.3%
	Prefer not to answer	48	18.8%

Detailed sociodemographic distributions are provided in Supplementary Tables 1, 2.

### Scale scores, level classifications, and internal consistency

The main study constructs were concentrated toward high scores. Anticipated behavior toward future epidemics had the highest mean score (*M* = 4.361, SD = 0.728), followed by moral perceptions (*M* = 3.947, SD = 0.630) and public trust (*M* = 3.903, SD = 0.651). The total scale score was also high (*M* = 4.071, SD = 0.599). High-level classification was the dominant pattern for anticipated behavior (88.7%), moral perceptions (76.2%), and public trust (75.4%). Internal consistency was acceptable to excellent across the scales, with Cronbach’s alpha values of 0.773 for public trust, 0.706 for moral perceptions, 0.886 for anticipated behavior, and 0.912 for the total scale (Table 2; Figures 1, 2).

**Table 2:** Scale descriptives, level classifications, and internal consistency.

Scale	Mean ± SD	Median (IQR)	Range	Low n (%)	Moderate n (%)	High n (%)	Cronbach’s α
Public trust	3.903 ± 0.651	4.200 (3.700–4.200)	1.700–5.000	12 (4.7%)	51 (19.9%)	193 (75.4%)	0.773
Moral perceptions	3.947 ± 0.630	3.900 (3.700–4.400)	2.000–5.000	5 (2.0%)	56 (21.9%)	195 (76.2%)	0.706
Anticipated behavior	4.361 ± 0.728	4.600 (4.200–4.800)	1.000–5.000	8 (3.1%)	21 (8.2%)	227 (88.7%)	0.886
Total scale	4.071 ± 0.599	4.200 (3.867–4.467)	1.667–5.000	–	–	–	0.912

IQR, interquartile range. Scale-level classifications were calculated using the coded scale cutoffs applied in the results dataset.

**Figure 1:**
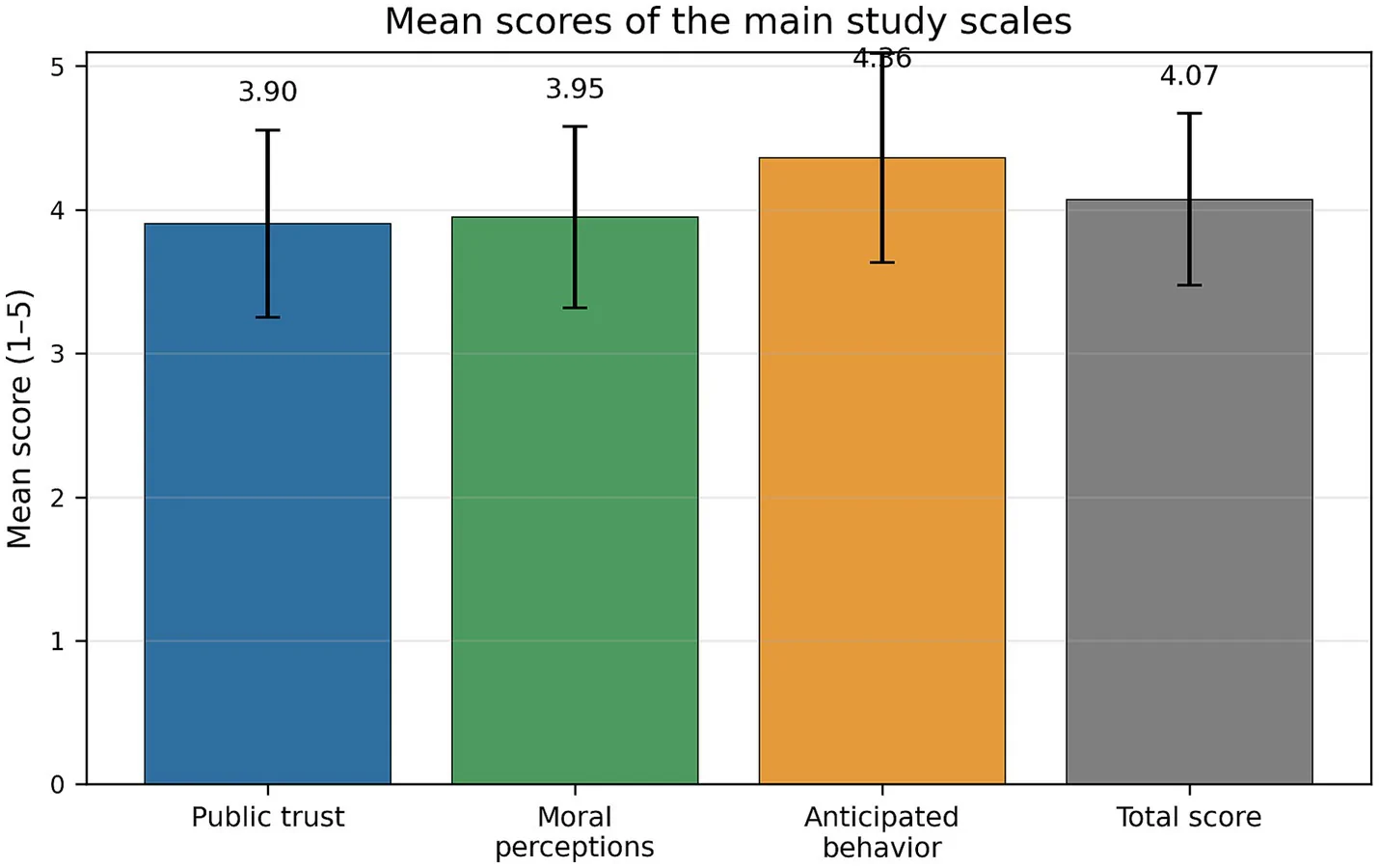
Mean scores of the main study scales with standard deviation bars.

**Figure 2:**
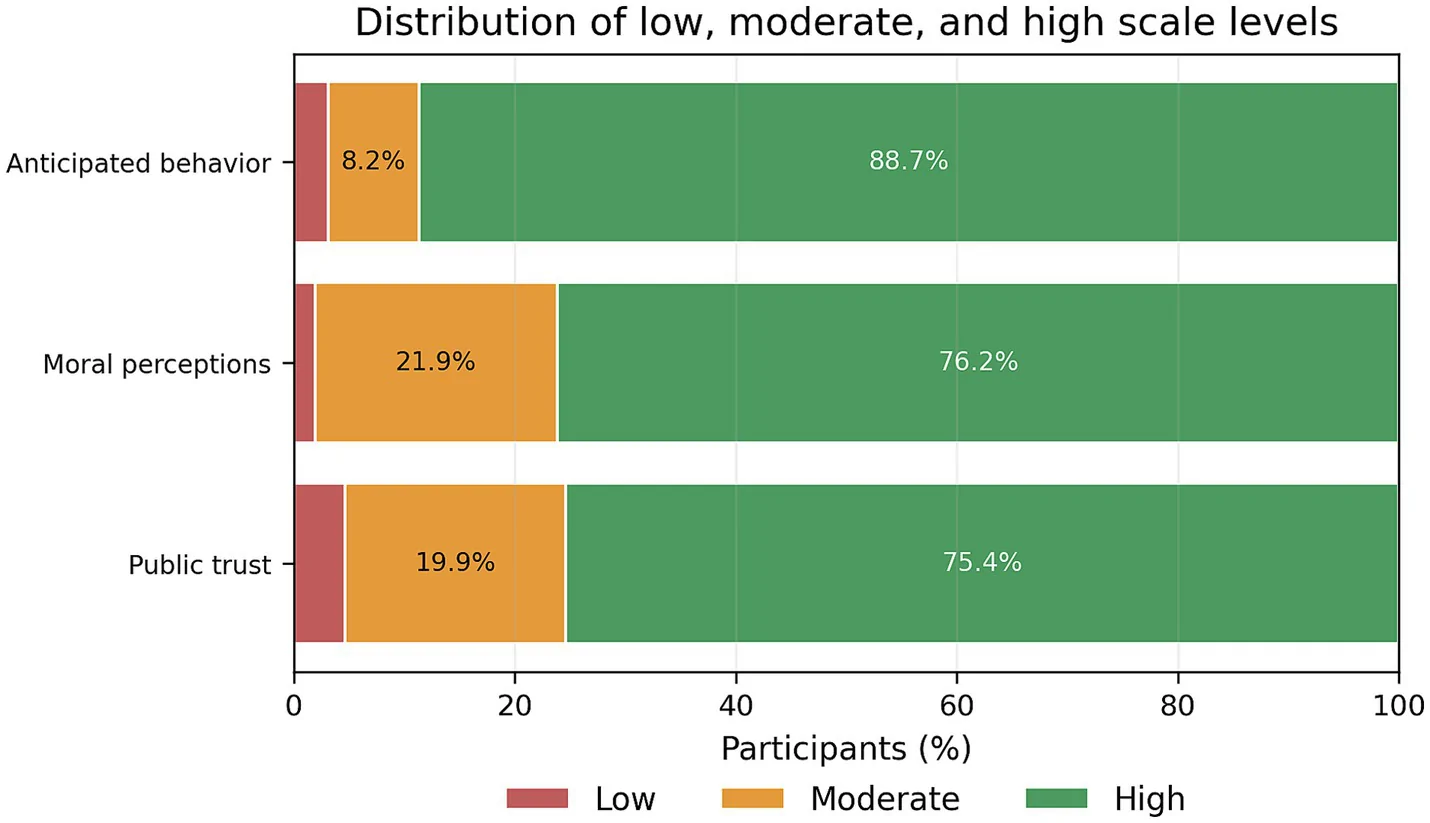
Distribution of low, moderate, and high levels across the three main constructs.

### Item-level response patterns

Item-level results showed strong endorsement of Saudi institutional trust, responsibility-oriented moral reasoning, and future preventive behavior. In the public trust section, the highest item was the belief that Saudi health authorities mainly act in the public’s health interests (T7: *M* = 4.54, SD = 0.96; 73.4% strongly agree). In the moral perceptions section, the strongest item was that people should follow health recommendations when these recommendations are linked to protecting others (M10: *M* = 4.61, SD = 0.82; 73.8% strongly agree), followed by the view that spreading unverified health information during epidemics is morally unacceptable (M9: *M* = 4.54, SD = 1.01; 77.3% strongly agree). In the anticipated behavior section, direct preventive behaviors were strongly endorsed, but willingness to receive recommended vaccines in future epidemics (B5) was comparatively lower (*M* = 4.17, SD = 1.20; 59.4% strongly agree), with 23.0% of respondents being neutral or disagreeing (Table 3; Figures 3–5; Supplementary Figure A7).

**Table 3:** Key item-level signals from the three questionnaire sections.

Construct	Key high-endorsed item	Mean ± SD	Strongly agree	Lower/critical item	Mean ± SD	Key interpretation
Public trust	T7. Saudi health authorities mainly act in the public’s health interests	4.54 ± 0.96	188 (73.4%)	T8. WHO mainly acts in the public’s health interests	3.83 ± 1.35	Trust was strong for Saudi institutions and comparatively less consistent for international trust.
Moral perceptions	M10. Follow recommendations when linked to protecting others	4.61 ± 0.82	189 (73.8%)	M8_R. Ignoring official guidance can be morally acceptable in some situations	2.52 ± 1.56	Collective responsibility was stronger than permissive attitudes toward ignoring guidance.
Anticipated behavior	B2. Maintain hand hygiene and clean frequently touched surfaces	4.63 ± 0.88	203 (79.3%)	B5. Receive recommended vaccines in future epidemics	4.17 ± 1.20	Vaccine willingness remained positive but was the clearest behavior-specific vulnerability.

Full five-point response distributions for all items are provided in Supplementary Tables 3–5. Reverse-coded items are marked with _R.

**Figure 3:**
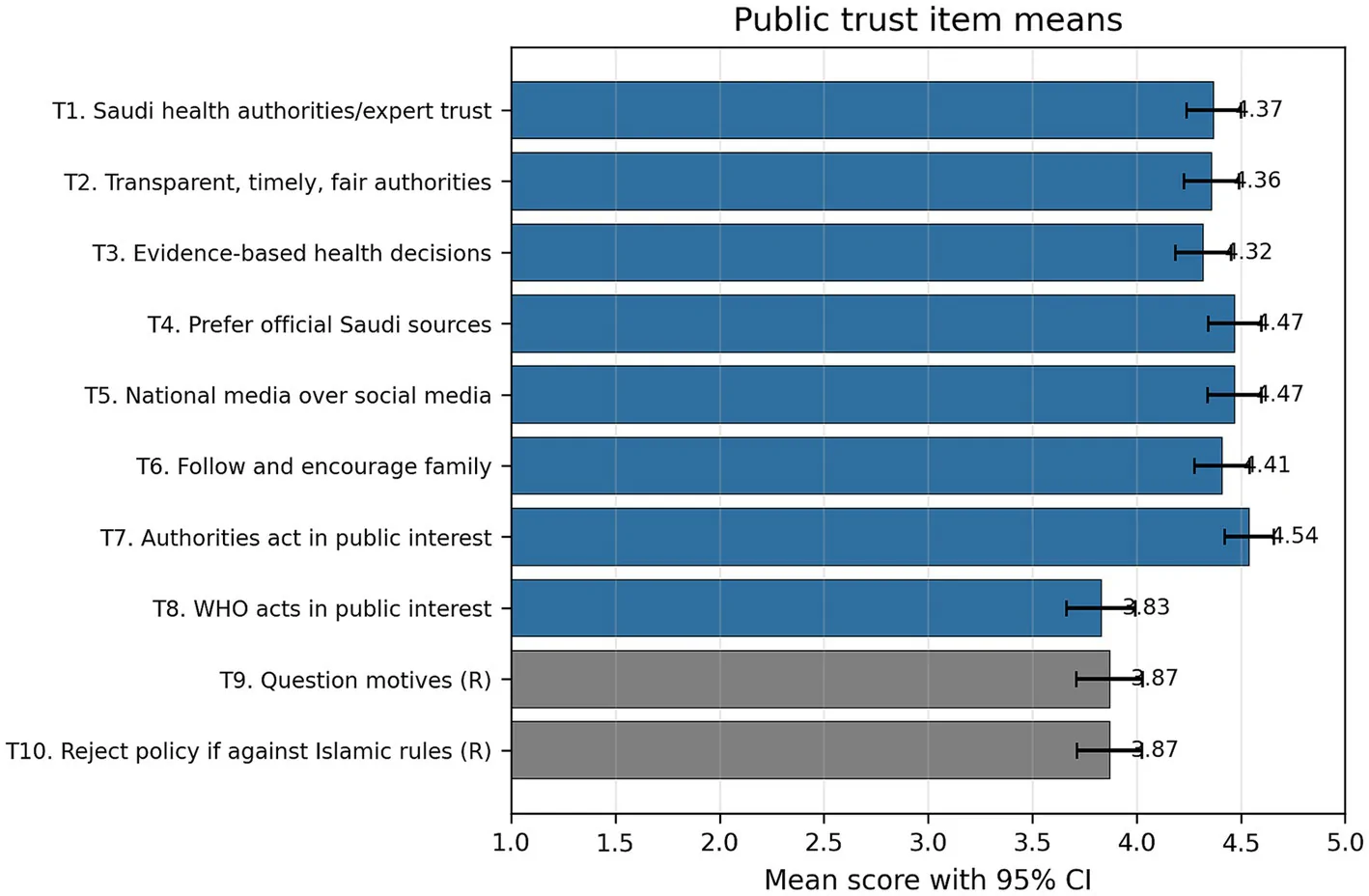
Public trust item means with 95% confidence intervals.

**Figure 4:**
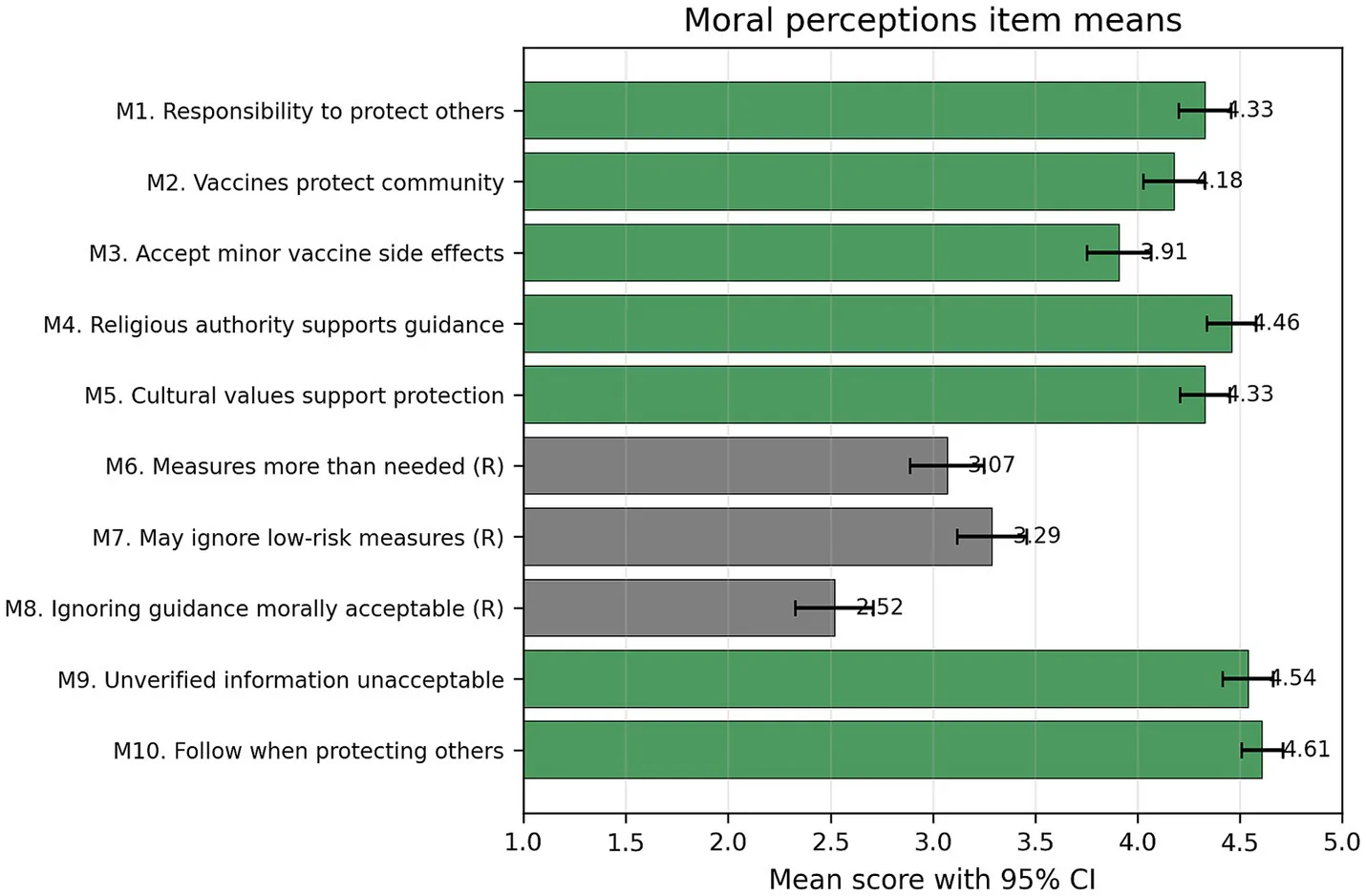
Moral perception item means with 95% confidence intervals.

**Figure 5:**
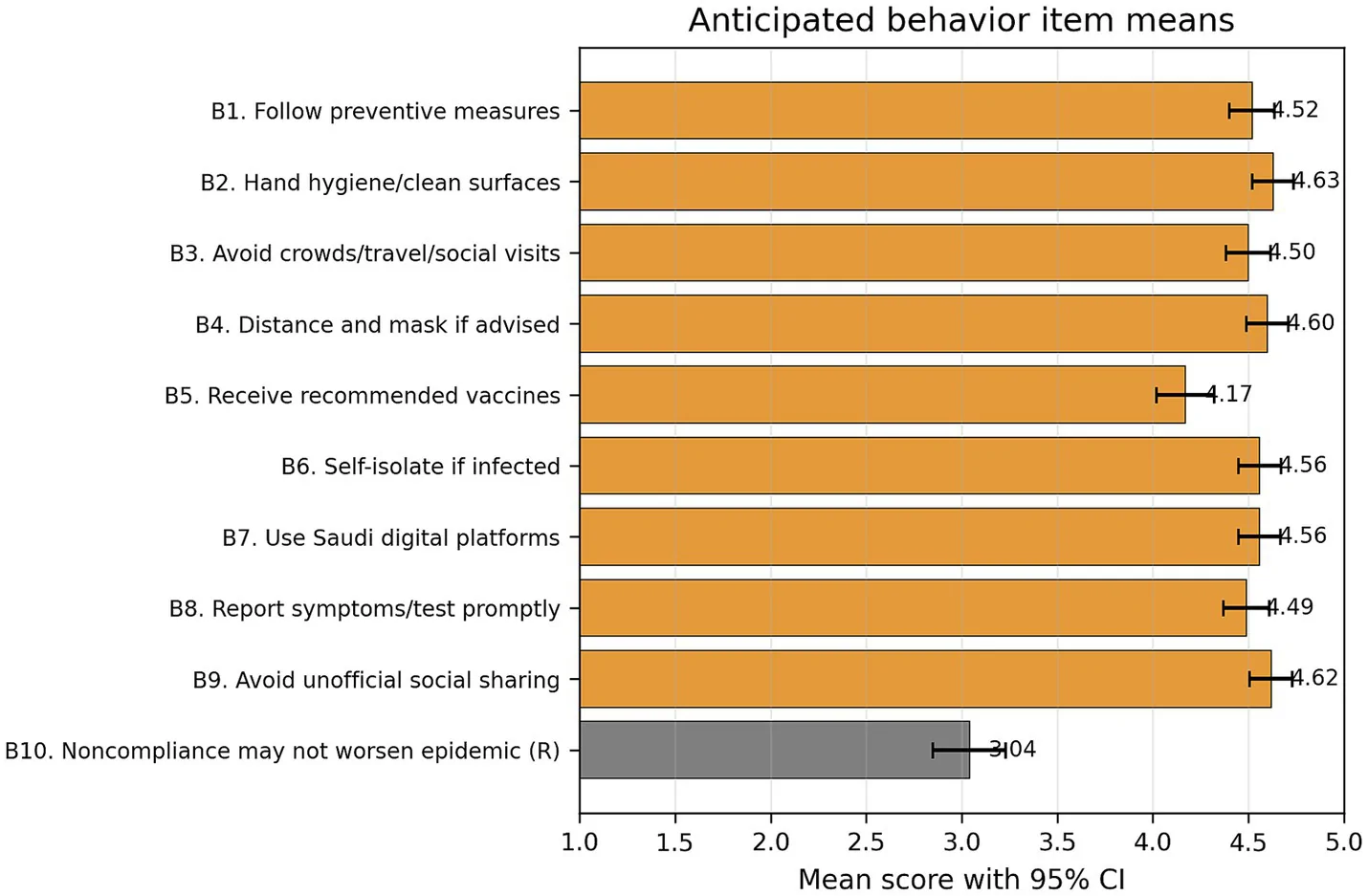
Anticipated behavior item means with 95% confidence intervals.

### Bivariate relationships and subgroup differences

Bivariate analysis supported the expected relationships among the core constructs. Anticipated behavior was positively correlated with public trust (*r* = 0.671, *p* < 0.001) and moral perceptions (*r* = 0.742, *p* < 0.001), and moral perceptions were also positively correlated with public trust (*r* = 0.677, *p* < 0.001). Age showed only a small positive relationship with anticipated behavior (*r* = 0.190, *p* = 0.002). Group comparisons indicated that anticipated behavior differed by gender (*p* = 0.049), marital status (*p* < 0.001), education level (*p* < 0.001), and employment status (*p* = 0.017), while household income was not significantly associated with anticipated behavior (*p* = 0.161). When anticipated behavior was analyzed as a low/moderate/high classification, public trust level and moral perception level were strongly associated with anticipated behavior level (both *p* < 0.001), showing a clear graded pattern (Table 4; Figures 6, 7).

**Table 4:** Main bivariate and subgroup findings for anticipated behavior.

Analysis domain	Predictor/factor	Result for anticipated behavior	*p*-value	Interpretation
Pearson correlation	Public trust	*r* = 0.671	<0.001	Higher trust was strongly associated with greater anticipated adherence.
Pearson correlation	Moral perceptions	*r* = 0.742	<0.001	Moral perceptions showed the strongest bivariate relationship with anticipated behavior.
Pearson correlation	Age	*r* = 0.190	0.002	Age had a small positive association with anticipated behavior.
Gender comparison	Male vs. female	Male: 4.288 ± 0.828; Female: 4.472 ± 0.531	0.049	Females had a higher mean anticipated behavior score.
ANOVA	Marital status	*F*(3,252) = 7.845	<0.001	Anticipated behavior differed across marital groups.
ANOVA	Education level	*F*(4,251) = 6.628	<0.001	Anticipated behavior differed across education groups.
ANOVA	Employment status	*F*(9,246) = 2.192	0.017	Anticipated behavior differed across employment categories.
ANOVA	Household income	*F*(4,203) = 1.658	0.161	No significant income-based difference was observed.
χ^2^ association	Public trust level	χ^2^(4) = 68.280	<0.001	High trust corresponded to a much larger high-behavior proportion.
χ^2^ association	Moral perception level	χ^2^(4) = 147.222	<0.001	The graded pattern was especially strong for moral perceptions.

Full Pearson and Spearman matrices, gender comparisons, ANOVA summaries, and categorical association tables are provided in Supplementary Tables 11–16.

**Figure 6:**
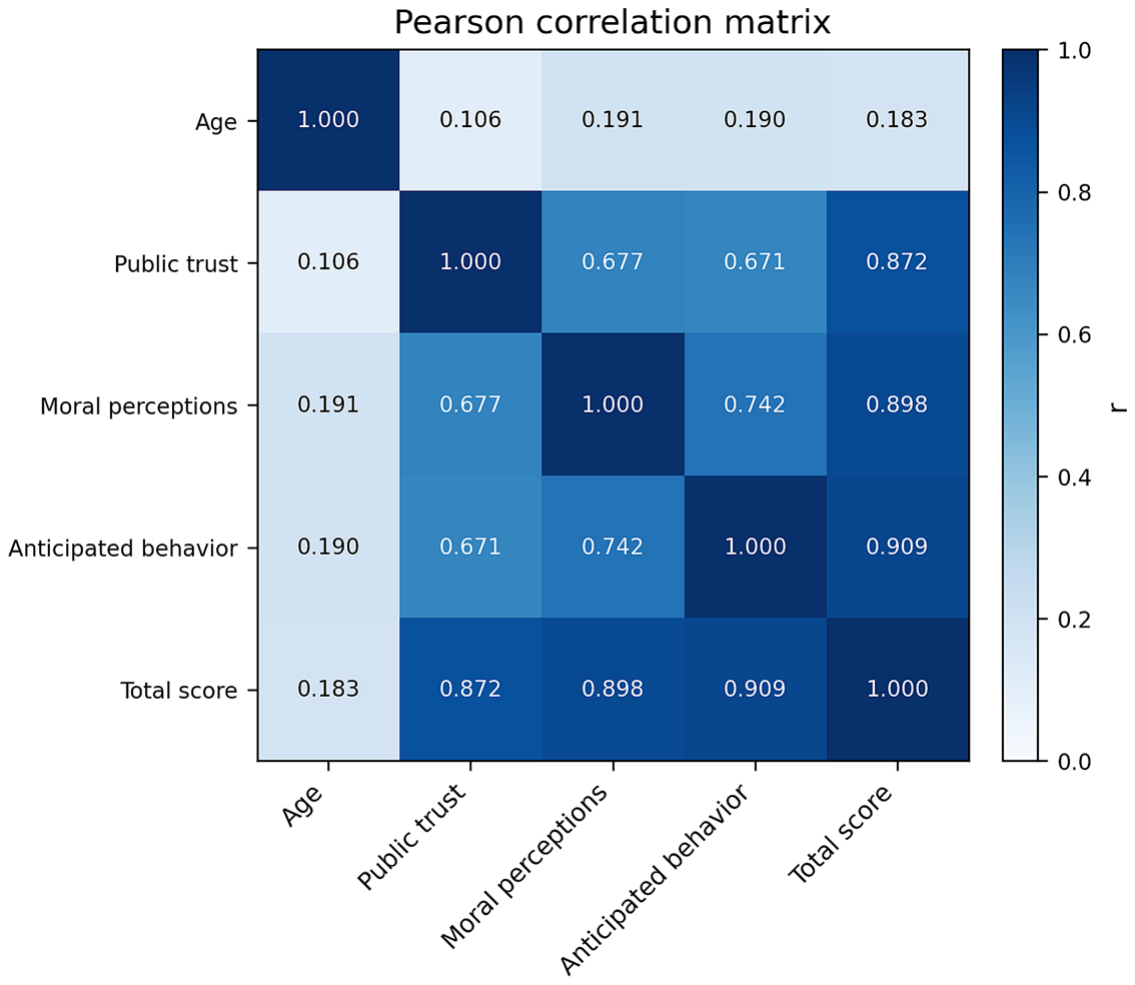
Pearson correlation heatmap for age and the main study scores.

**Figure 7:**
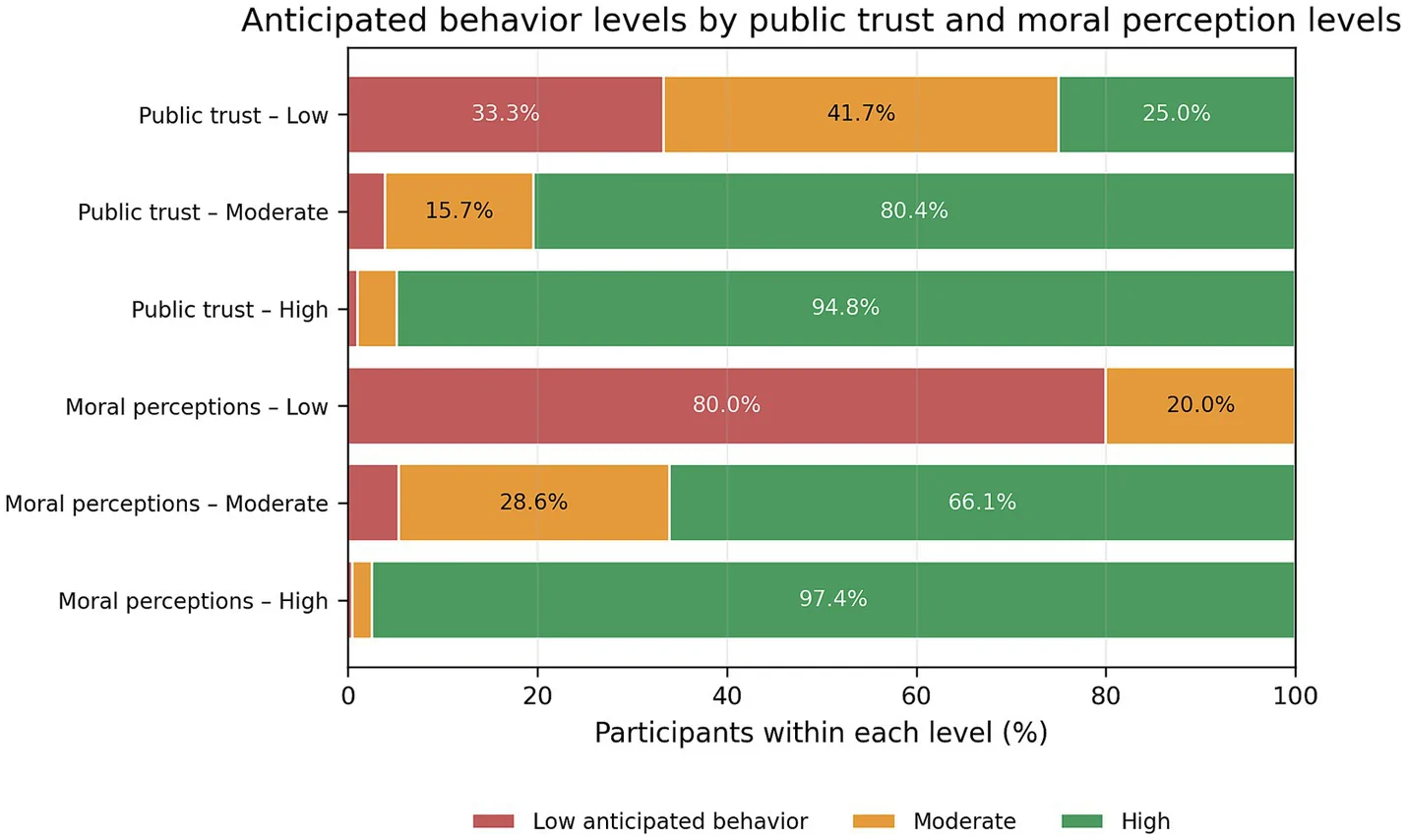
Anticipated behavior levels by public trust and moral perception levels.

### Multivariable predictors of anticipated behavior

The primary two-predictor regression model was statistically significant, *F*(2,253) = 192.693, *p* < 0.001, explaining 60.4% of anticipated-behavior variance (*R*^2^ = 0.604; adjusted *R*^2^ = 0.601). Public trust (*B* = 0.348, SE = 0.060, standardized beta = 0.311, 95% CI 0.229–0.466, *p* < 0.001) and moral perceptions (*B* = 0.614, SE = 0.062, standardized beta = 0.532, 95% CI 0.492–0.737, *p* < 0.001) were both positively associated with anticipated behavior. VIF was 1.847 and tolerance was 0.541 for both predictors, indicating no problematic multicollinearity; the Durbin-Watson statistic was 1.880. The maximum Cook’s distance was 0.198 in the primary model and 0.283 in the fully adjusted model, and no observation exceeded Cook’s *D* = 1. Twenty observations exceeded the conservative 4/N screening threshold in the fully adjusted model. After excluding these observations in a sensitivity analysis (*N* = 236), both public trust (*B* = 0.384, 95% CI 0.291–0.476, *p* < 0.001) and moral perceptions (*B* = 0.401, 95% CI 0.313–0.489, *p* < 0.001) remained significant.

## Result synthesis

The full-sample adjusted model, which retained income non-disclosure as a separate response category, was statistically significant, *F*(25,230) = 20.590, *p* < 0.001, with *R*^2^ = 0.691 and adjusted *R*^2^ = 0.658. Public trust remained positively associated with anticipated behavior (*B* = 0.357, 95% CI 0.244–0.470, standardized beta = 0.319, *p* < 0.001), as did moral perceptions (*B* = 0.495, 95% CI 0.374–0.615, standardized beta = 0.428, *p* < 0.001). Household income was not significant as an overall adjusted factor (*p* = 0.419). Residual-versus-fitted and Q-Q plots showed heteroscedasticity and some tail departure, and the Breusch-Pagan test was significant (*p* < 0.001). HC3 robust estimation did not change the conclusions: public trust (*B* = 0.357, HC3 SE = 0.079, 95% CI 0.201–0.512, *p* < 0.001) and moral perceptions (*B* = 0.495, HC3 SE = 0.061, 95% CI 0.374–0.615, *p* < 0.001) remained significant. Collectively, the conventional, robust, and influence-sensitivity analyses supported the main hypothesis (Figure 8).

**Figure 8:**
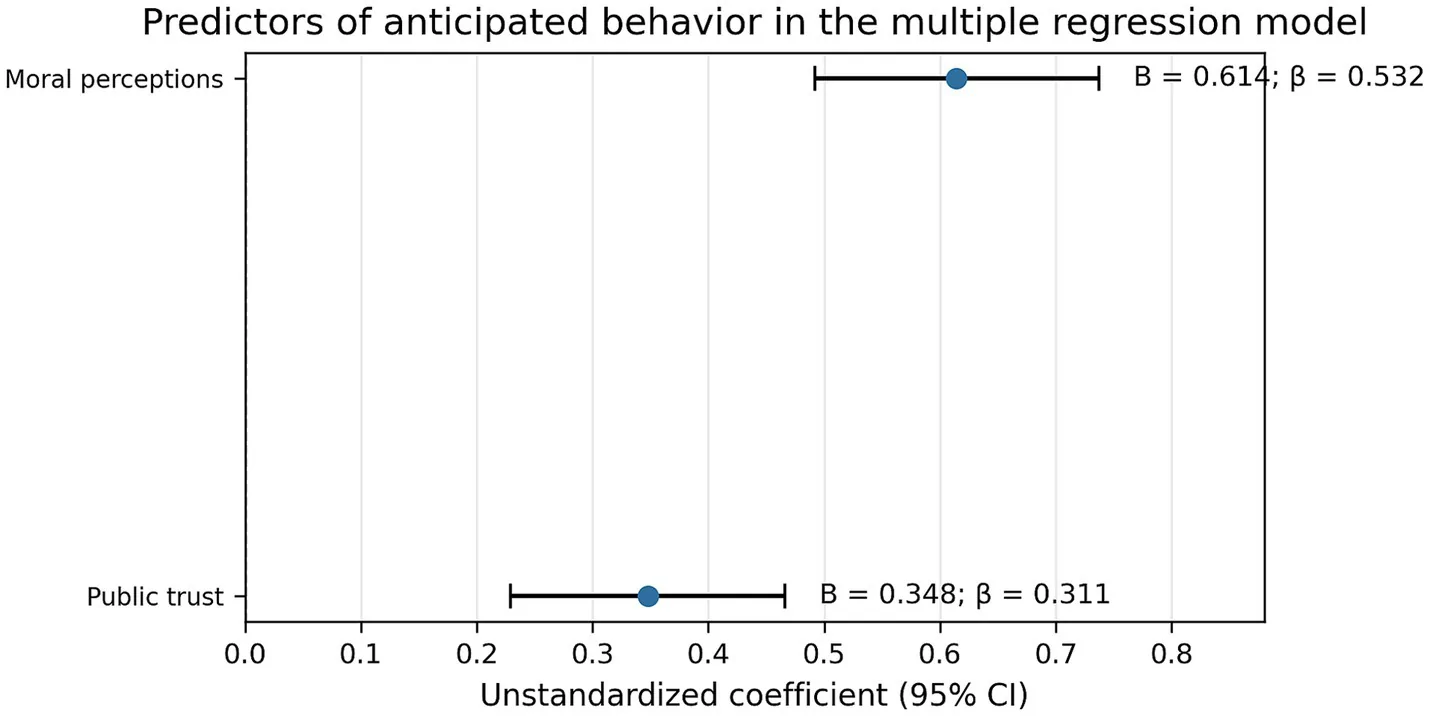
Regression coefficients predicting anticipated behavior in the multiple linear regression model.

## Discussion

The present study examined public trust, moral perceptions, and anticipated behavioral responses toward future epidemics among 256 adults. Anticipated behavior was generally high, with a mean score of 4.361 and 88.7% of participants classified at the high level. Public trust and moral perceptions were also high, with high-level classifications in 75.4 and 76.2% of participants, respectively. The results indicated strong self-reported readiness to follow future epidemic control measures, although readiness varied across behavioral domains and sociodemographic groups. This pattern was consistent with Saudi evidence of high reliance on official sources and substantial adoption of preventive practices during COVID-19 (6, 21).

Public trust was an important finding because institutional credibility can support voluntary cooperation when emergency guidance changes or requires repeated personal sacrifice. Participants expressed particularly strong confidence that Saudi health authorities act in the public’s health interests. This finding is consistent with cross-national evidence linking trust with perceptions of organized, fair, clearly communicated responses and with the maintenance of protective behavior over time (11, 12). However, trust was not uniform across all sources. Comparatively lower scores for the World Health Organization item and reverse-coded skepticism items show that high national trust can coexist with selective doubts. Future communication must therefore preserve credibility through transparency, prompt correction of misinformation, and clear explanations of the scientific and ethical basis for recommendations (7, 8).

Moral perceptions showed an especially strong role. Participants endorsed the responsibility to follow recommendations that protect others and viewed the spread of unverified health information as morally unacceptable. This suggests that anticipated adherence reflected not only respect for authority or fear of illness but also a perceived duty to prevent harm. The finding accords with evidence that social norms, collective identity, moral reasoning, and concern for vulnerable people influence pandemic behavior (1, 13). In the Saudi context, such responsibility-based reasoning may resonate with cultural and religious values that emphasize protection of family and community (Table 5).

**Table 5:** Regression and adjusted model results for anticipated behavior.

Model/component	Predictor/statistic	Estimate	95% CI/df	*p*-value	Interpretation
Primary regression	Model fit	*R* = 0.777; *R*^2^ = 0.604; adjusted *R*^2^ = 0.601	*F*(2,253) = 192.693	<0.001	Core predictors explained 60.4% of variance.
Primary regression	Public trust	*B* = 0.348; SE = 0.060; beta = 0.311	0.229–0.466	<0.001	Positive adjusted association.
Primary regression	Moral perceptions	*B* = 0.614; SE = 0.062; beta = 0.532	0.492–0.737	<0.001	Stronger positive association.
Full-sample adjusted model	Model fit	*N* = 256; *R*^2^ = 0.691; adjusted *R*^2^ = 0.658	*F*(25,230) = 20.590	<0.001	Income non-disclosure retained as a response category.
Full-sample adjusted model	Public trust	*B* = 0.357; beta = 0.319	0.244–0.470	<0.001	Remained significant after adjustment.
Full-sample adjusted model	Moral perceptions	*B* = 0.495; beta = 0.428	0.374–0.615	<0.001	Stronger adjusted association.
Full-sample adjusted model	Household income (overall)	–	–	0.419	Not statistically significant overall.
HC3 robust model	Public trust	*B* = 0.357; robust SE = 0.079	0.201–0.512	<0.001	Conclusion unchanged.
HC3 robust model	Moral perceptions	*B* = 0.495; robust SE = 0.061	0.374–0.615	<0.001	Conclusion unchanged.
Influence sensitivity	After excluding 20 flagged observations	*N* = 236	Public trust: 0.291–0.476; moral perceptions: 0.313–0.489	Both <0.001	Both associations remained significant.

HC3 = heteroscedasticity-consistent standard errors, version 3. The 4/N Cook’s-distance value was used as a screening threshold rather than an automatic exclusion rule.

The regression analyses supported the central hypothesis. Public trust and moral perceptions explained 60.4% of anticipated-behavior variance in the primary model, with moral perceptions showing the larger standardized association, and both remained significant in the fully adjusted *N* = 256 model. HC3 robust standard errors and influence-sensitivity analyses did not materially change the conclusions, indicating that the associations were not explained by conventional standard-error assumptions or a small number of influential observations. Nevertheless, the cross-sectional design supports interpretation as adjusted association rather than causation (Tables 6, 7).

**Table 6:** Structure and scoring of the study measures.

Construct/section	Items	Score range	Direction of scoring	Reverse-coded items
Public trust	10	1–5	Higher = greater trust	T9, T10
Moral perceptions	10	1–5	Higher = stronger moral responsibility	M6, M7, M8
Anticipated behavior	10	1–5	Higher = stronger anticipated adherence	B10
Overall total score	30	1–5	Higher = stronger overall profile	All relevant reverse-coded items

**Table 7:** Internal consistency reliability of the study scales.

Scale	Number of items	Cronbach’s alpha
Public trust	10	0.773
Moral perceptions	10	0.706
Anticipated behavior	10	0.886
Total scale	30	0.912

Behavior-specific results qualified the high composite score. Anticipated adherence was strong for hand hygiene, cleaning, distancing, mask use, self-isolation, symptom reporting, and official digital health platforms, consistent with previous Saudi evidence (6, 10). Vaccine willingness was more fragile: although its mean remained positive at 4.17, 23.0% of participants were neutral or disagreed with future recommended vaccination. Saudi, international, and regional studies identify vaccine safety concerns, mistrust in institutions or approval processes, misinformation, and conspiracy beliefs as recurring barriers (14–19, 22). The present item referred to vaccines recommended in future epidemics rather than a specific COVID-19 vaccine, which may partly explain its higher score relative to some regional studies. Taken together, these findings show that high general readiness should not be interpreted as uniform acceptance and that vaccine communication should integrate transparent safety information, institutional trust-building, and responsibility toward others.

Subgroup analyses indicated variation by gender, marital status, education, and employment, whereas household income was not significant overall in the full-sample adjusted model. The 48 participants who preferred not to disclose income were retained as a separate response category, preserving the full sample without treating non-disclosure as an income level. These subgroup patterns identify populations for further study and tailored communication, but they should be interpreted cautiously because some categories were small and the sampling design was non-probabilistic.

The findings are consistent with a behavioral framework in which attitudes toward official guidance and perceived social or moral norms jointly shape intended adherence (9). Saudi Arabia’s experience with MERS-CoV and COVID-19 also provides an institutional and sociocultural context in which trust, public communication, perceived risk, and communal responsibility may interact (4–6). Future preparedness models should therefore integrate credible governance and communication with behavioral science rather than treating compliance as a uniform response to information alone.

Overall, anticipated behavior toward future epidemics was strongly associated with both institutional trust and moral responsibility, with moral perceptions showing the larger independent association. The results extend Saudi evidence beyond behavior during a specific phase of COVID-19 to future-oriented readiness. They also show that preparedness strategies should combine credible institutional communication with ethical messages about community protection while addressing behavior-specific vulnerabilities, particularly vaccination.

### Implications

These findings support several concrete actions for epidemic preparedness in Saudi Arabia. First, the Ministry of Health and other official communicators could prepare a coordinated message framework for future outbreaks in which each recommendation is accompanied by a brief explanation of the evidence, known uncertainties, expected benefit, and the specific action required from the public. Second, official digital channels could publish synchronized Arabic updates, rapidly correct circulating misinformation, and maintain a publicly accessible question-and-answer log as guidance evolves. Third, vaccination should have a dedicated communication pathway that explains approval and safety monitoring, distinguishes established evidence from uncertainty, and uses trusted clinicians together with appropriate community and religious leaders to address concerns without stigmatizing hesitant groups. Fourth, messages should be pretested with groups showing more variable anticipated adherence, including people with lower educational attainment and non-healthcare occupational groups, and adapted for readability and cultural relevance. Finally, preparedness programs should monitor trust, rumor exposure, message comprehension, vaccine confidence, and behavior-specific intentions over time so that communication can be adjusted before gaps in adherence widen.

### Recommendations

Future research should evaluate these interventions prospectively and distinguish between specific preventive behaviors rather than relying only on an overall adherence score. Longitudinal and experimental studies could test whether transparent uncertainty communication, moral framing, and trusted-messenger strategies improve vaccination, isolation, masking, and other behaviors separately. Probability-based sampling across multiple Saudi regions and inclusion of risk perception, prior infection, and religiosity would also improve causal interpretation, generalizability, and control of confounding.

### Limitations

This study had several limitations. Its cross-sectional design precluded causal inference; all reported relationships should therefore be interpreted as associations. Self-reported responses may have been affected by recall, social-desirability, and common-method bias, particularly for morally valued behaviors. The outcome represented anticipated rather than observed behavior, and intentions may differ from actions during a future epidemic. Convenience and snowball recruitment, online distribution, and restriction to adults in Riyadh may have introduced selection bias and limit generalizability. No separate pilot study was conducted, although translation, expert content-validity assessment, internal-consistency analysis, and item diagnostics were undertaken. The concentration of anticipated-behavior scores near the upper end of the scale produced heteroscedasticity and some residual-tail departure; however, HC3 robust and influence-sensitivity analyses yielded unchanged conclusions. Some demographic categories were small. In addition, the model did not include individual risk perception, prior infection, or religiosity, all of which may influence compliance behavior; residual confounding by these unmeasured factors therefore remains possible. The anticipated-behavior outcome also aggregated 10 distinct behaviors into a single mean score. Because determinants may differ across vaccination, isolation, masking, hygiene, and other measures, this aggregation may obscure behavior-specific associations, particularly the greater fragility observed for vaccine willingness. Finally, 18.8% of participants did not disclose household income. Treating non-disclosure as a separate response category preserved the full sample but did not recover the underlying income value; accordingly, income-related findings should be interpreted cautiously.

## Conclusion

This study showed that adults in the sample had high public trust, strong responsibility-oriented moral perceptions, and high anticipated adherence to preventive behaviors during future epidemics. Public trust and moral perceptions were both positively associated with anticipated behavior, and both remained significant independent predictors in the regression analysis, with moral perceptions showing the stronger contribution. The findings suggested that future epidemic preparedness in Saudi Arabia would benefit from communication strategies that protect institutional credibility while also emphasizing the ethical and community-protection reasons for adherence. Although overall readiness was high, the relatively greater uncertainty around future vaccination and selected skepticism-related items indicated that preparedness efforts should not assume uniform compliance across all preventive behaviors. The evidence supported a prevention model in which trust, moral responsibility, targeted communication, and misinformation control worked together to strengthen population readiness for future health emergencies.

## Data Availability

The raw data supporting the conclusions of this article will be made available by the authors, without undue reservation.
