# Supplementary material for: Public trust and moral perceptions as predictors of anticipated preventive behavior toward future epidemics among adults in Riyadh, Saudi Arabia: a cross-sectional study
Source: Front Public Health. 2026 Oct 7;14:1950414. doi: 10.3389/fpubh.2026.1950414 (PMC13685596; doi:10.3389/fpubh.2026.1950414)
Supplement: Supplementary file 1 [file Data_sheet_1.docx]

# Supplementary Material

Detailed tables and supplementary figures referenced in the main manuscript are provided below.

**Appendix: Detailed Results Tables and Supplementary Figures**

The appendix keeps the detailed outputs that were condensed or not displayed in the manuscript-ready results section. Tables are renumbered as Appendix Tables A1–A18 and figures as Appendix Figures A1–A7.

## Appendix allocation map

| **Main-text item** | **Source detail retained in appendix** |
| --- | --- |
| Table 1. Condensed analytic sample and participant characteristics | Appendix Tables A1–A2 |
| Table 2. Scale descriptives, level classifications, and reliability | Appendix Tables A6–A8 |
| Table 3. Key item-level signals | Appendix Tables A3–A5 |
| Table 4. Bivariate and subgroup findings | Appendix Tables A11–A16 |
| Table 5. Regression and adjusted model results | Appendix Tables A17–A18 |
| Main figures | Appendix Figures A1–A7 retain detailed or unused visual outputs. |

## Detailed tables

**Appendix Table A1. Data Quality and Analytic Sample Summary (N = 256)**

| **Indicator** | **n** | **%** |
| --- | --- | --- |
| Total valid survey responses | 256 | 100.0% |
| Valid Likert responses after cleaning | 256 | 100.0% |

**Appendix Table A2. Sociodemographic Characteristics of Participants (N = 256)**

| **Variable** | **Category / Statistic** | **n or value** | **% / range** |
| --- | --- | --- | --- |
| Age, years | Mean ± SD | 35.69 ± 10.10 | Range: 18–72 |
| Gender | Male | 154 | 60.2% |
|  | Female | 102 | 39.8% |
| Marital status | Single / unmarried | 95 | 37.1% |
|  | Married | 150 | 58.6% |
|  | Divorced | 6 | 2.3% |
|  | Widowed | 5 | 2.0% |
| Education level | Less than high school | 11 | 4.3% |
|  | High school diploma | 41 | 16.0% |
|  | Bachelor's degree | 141 | 55.1% |
|  | Master's degree | 45 | 17.6% |
|  | Doctoral degree | 18 | 7.0% |
| Employment status | Government employee (civilian) | 46 | 18.0% |
|  | Healthcare worker | 55 | 21.5% |
|  | Education sector | 19 | 7.4% |
|  | Private sector employee | 32 | 12.5% |
|  | Self-employed / freelancer | 13 | 5.1% |
|  | Military / security sector | 16 | 6.2% |
|  | Housewife | 16 | 6.2% |
|  | Student | 32 | 12.5% |
|  | Retired | 13 | 5.1% |
|  | Unemployed / looking for work | 14 | 5.5% |
| Household monthly income | Less than 3,000 SAR | 20 | 7.8% |
|  | 3,000-6,000 SAR | 22 | 8.6% |
|  | 6,001-10,000 SAR | 24 | 9.4% |
|  | 10,001-15,000 SAR | 49 | 19.1% |
|  | More than 15,000 SAR | 93 | 36.3% |
|  | Prefer not to answer | 48 | 18.8% |

**Appendix Table A3. Public Trust Items: Response Distribution and Descriptive Statistics (N = 256)**

| **Item** | **Statement** | **Strongly disagree n (%)** | **Disagree n (%)** | **Neutral n (%)** | **Agree n (%)** | **Strongly agree n (%)** | **Mean ± SD** |
| --- | --- | --- | --- | --- | --- | --- | --- |
| T1 | Trust Saudi health authorities and health experts to manage future epidemics effectively | 13 (5.1%) | 5 (2.0%) | 22 (8.6%) | 50 (19.5%) | 166 (64.8%) | 4.37 ± 1.07 |
| T2 | Belief that Saudi health authorities are transparent, timely, and fair during health emergencies | 12 (4.7%) | 9 (3.5%) | 21 (8.2%) | 46 (18.0%) | 168 (65.6%) | 4.36 ± 1.08 |
| T3 | Belief that health decisions in Saudi Arabia are based on scientific evidence | 13 (5.1%) | 7 (2.7%) | 27 (10.5%) | 46 (18.0%) | 163 (63.7%) | 4.32 ± 1.10 |
| T4 | Preference for official Saudi sources when social media information differs | 12 (4.7%) | 6 (2.3%) | 15 (5.9%) | 40 (15.6%) | 183 (71.5%) | 4.47 ± 1.04 |
| T5 | Trust official national media more than social media for epidemic-related information | 12 (4.7%) | 7 (2.7%) | 15 (5.9%) | 37 (14.5%) | 185 (72.3%) | 4.47 ± 1.05 |
| T6 | Willingness to follow public health instructions and encourage family to do the same | 12 (4.7%) | 8 (3.1%) | 21 (8.2%) | 37 (14.5%) | 178 (69.5%) | 4.41 ± 1.08 |
| T7 | Belief that Saudi health authorities mainly act in the public’s health interests | 10 (3.9%) | 5 (2.0%) | 11 (4.3%) | 42 (16.4%) | 188 (73.4%) | 4.54 ± 0.96 |
| T8 | Belief that the WHO mainly acts in the public’s health interests | 24 (9.4%) | 22 (8.6%) | 46 (18.0%) | 45 (17.6%) | 119 (46.5%) | 3.83 ± 1.35 |
| T9 | Caution and questioning motives behind some health recommendations (reverse-coded) | 18 (7.0%) | 30 (11.7%) | 37 (14.5%) | 53 (20.7%) | 118 (46.1%) | 3.87 ± 1.30 |
| T10 | Acceptability of not following policy if it clearly contradicts Islamic rules (reverse-coded) | 20 (7.8%) | 17 (6.6%) | 57 (22.3%) | 44 (17.2%) | 118 (46.1%) | 3.87 ± 1.28 |

*Note. Full five-point Likert distributions are retained here; abbreviated key findings are reported in the main manuscript results section.*

**Appendix Table A4. Moral Perception Items: Response Distribution and Descriptive Statistics (N = 256)**

| **Item** | **Statement** | **Strongly disagree n (%)** | **Disagree n (%)** | **Neutral n (%)** | **Agree n (%)** | **Strongly agree n (%)** | **Mean ± SD** |
| --- | --- | --- | --- | --- | --- | --- | --- |
| M1 | Personal responsibility for protecting others during health emergencies | 10 (3.9%) | 7 (2.7%) | 28 (10.9%) | 55 (21.5%) | 156 (60.9%) | 4.33 ± 1.04 |
| M2 | Taking recommended vaccines as a responsible action to protect the community | 17 (6.6%) | 14 (5.5%) | 28 (10.9%) | 43 (16.8%) | 154 (60.2%) | 4.18 ± 1.22 |
| M3 | Acceptance of minor vaccine side effects to protect people at higher risk | 23 (9.0%) | 14 (5.5%) | 43 (16.8%) | 59 (23.0%) | 117 (45.7%) | 3.91 ± 1.28 |
| M4 | Religious beliefs encourage following health guidance when supported by trusted religious authorities | 11 (4.3%) | 4 (1.6%) | 16 (6.2%) | 51 (19.9%) | 174 (68.0%) | 4.46 ± 0.99 |
| M5 | Saudi cultural values support accepting health interventions that protect others | 8 (3.1%) | 10 (3.9%) | 21 (8.2%) | 67 (26.2%) | 150 (58.6%) | 4.33 ± 1.00 |
| M6 | Some preventive measures are more than what is really needed (reverse-coded) | 55 (21.5%) | 43 (16.8%) | 51 (19.9%) | 43 (16.8%) | 64 (25.0%) | 3.07 ± 1.48 |
| M7 | May not follow every health measure if the actual risk is very low (reverse-coded) | 42 (16.4%) | 32 (12.5%) | 54 (21.1%) | 65 (25.4%) | 63 (24.6%) | 3.29 ± 1.39 |
| M8 | Ignoring official health guidance can be morally acceptable in some situations (reverse-coded) | 107 (41.8%) | 35 (13.7%) | 33 (12.9%) | 36 (14.1%) | 45 (17.6%) | 2.52 ± 1.56 |
| M9 | Spreading unverified health information during epidemics is morally unacceptable | 11 (4.3%) | 5 (2.0%) | 17 (6.6%) | 25 (9.8%) | 198 (77.3%) | 4.54 ± 1.01 |
| M10 | People should follow health recommendations when linked to protecting others | 7 (2.7%) | 1 (0.4%) | 11 (4.3%) | 48 (18.8%) | 189 (73.8%) | 4.61 ± 0.82 |

*Note. Full five-point Likert distributions are retained here; abbreviated key findings are reported in the main manuscript results section.*

**Appendix Table A5. Anticipated Behavior Items: Response Distribution and Descriptive Statistics (N = 256)**

| **Item** | **Statement** | **Strongly disagree n (%)** | **Disagree n (%)** | **Neutral n (%)** | **Agree n (%)** | **Strongly agree n (%)** | **Mean ± SD** |
| --- | --- | --- | --- | --- | --- | --- | --- |
| B1 | Willingness to follow preventive health measures and authority instructions in future epidemics | 11 (4.3%) | 3 (1.2%) | 13 (5.1%) | 44 (17.2%) | 185 (72.3%) | 4.52 ± 0.97 |
| B2 | Maintaining hand hygiene and cleaning frequently touched surfaces in epidemic situations | 8 (3.1%) | 4 (1.6%) | 9 (3.5%) | 32 (12.5%) | 203 (79.3%) | 4.63 ± 0.88 |
| B3 | Avoiding crowded places, unnecessary travel, and non-essential social visits during outbreaks | 9 (3.5%) | 4 (1.6%) | 17 (6.6%) | 46 (18.0%) | 180 (70.3%) | 4.50 ± 0.95 |
| B4 | Practicing social distancing and wearing a face mask if recommended | 8 (3.1%) | 4 (1.6%) | 11 (4.3%) | 36 (14.1%) | 197 (77.0%) | 4.60 ± 0.89 |
| B5 | Willingness to receive recommended vaccines in future epidemics | 20 (7.8%) | 9 (3.5%) | 30 (11.7%) | 45 (17.6%) | 152 (59.4%) | 4.17 ± 1.24 |
| B6 | Ability and willingness to self-isolate if infected | 7 (2.7%) | 5 (2.0%) | 17 (6.6%) | 36 (14.1%) | 191 (74.6%) | 4.56 ± 0.91 |
| B7 | Use of official Saudi digital health platforms during future epidemics | 6 (2.3%) | 5 (2.0%) | 20 (7.8%) | 34 (13.3%) | 191 (74.6%) | 4.56 ± 0.90 |
| B8 | Reporting symptoms or testing promptly if infection is suspected | 8 (3.1%) | 9 (3.5%) | 14 (5.5%) | 44 (17.2%) | 181 (70.7%) | 4.49 ± 0.97 |
| B9 | Avoiding sharing unofficial health information on social media during future outbreaks | 8 (3.1%) | 5 (2.0%) | 12 (4.7%) | 27 (10.5%) | 204 (79.7%) | 4.62 ± 0.91 |
| B10 | Future epidemics may not worsen even if some people do not fully follow guidance (reverse-coded) | 62 (24.2%) | 45 (17.6%) | 42 (16.4%) | 36 (14.1%) | 71 (27.7%) | 3.04 ± 1.55 |

*Note. Full five-point Likert distributions are retained here; abbreviated key findings are reported in the main manuscript results section.*

**Appendix Table A6. Scale-Level Descriptive Statistics**

| **Scale** | **N** | **Mean** | **SD** | **Minimum** | **25th percentile** | **Median** | **75th percentile** | **Maximum** |
| --- | --- | --- | --- | --- | --- | --- | --- | --- |
| Public trust | 256 | 3.903 | 0.651 | 1.700 | 3.700 | 4.200 | 4.200 | 5.000 |
| Moral perceptions | 256 | 3.947 | 0.630 | 2.000 | 3.700 | 3.900 | 4.400 | 5.000 |
| Anticipated behavior | 256 | 4.361 | 0.728 | 1.000 | 4.200 | 4.600 | 4.800 | 5.000 |
| Overall total score | 256 | 4.071 | 0.599 | 1.667 | 3.867 | 4.200 | 4.467 | 5.000 |

**Appendix Table A7. Distribution of Scale Level Classifications**

| **Scale** | **Level** | **n** | **%** |
| --- | --- | --- | --- |
| Public trust | Low | 12 | 4.7% |
|  | Moderate | 51 | 19.9% |
|  | High | 193 | 75.4% |
| Moral perceptions | Low | 5 | 2.0% |
|  | Moderate | 56 | 21.9% |
|  | High | 195 | 76.2% |
| Anticipated behavior | Low | 8 | 3.1% |
|  | Moderate | 21 | 8.2% |
|  | High | 227 | 88.7% |

**Appendix Table A8. Internal Consistency Reliability of the Study Scales**

| **Scale** | **Number of items** | **Cronbach’s alpha** |
| --- | --- | --- |
| Public trust | 10 | 0.773 |
| Moral perceptions | 10 | 0.706 |
| Anticipated behavior | 10 | 0.886 |
| Total scale | 30 | 0.912 |

**Appendix Table A9. Item-Total Reliability Diagnostics**

| **Scale** | **Item** | **Statement** | **Corrected item-total correlation** | **Cronbach’s alpha if item deleted** |
| --- | --- | --- | --- | --- |
| Public trust | T1 | Trust Saudi health authorities and health experts to manage future epidemics effectively | 0.725 | 0.717 |
|  | T2 | Belief that Saudi health authorities are transparent, timely, and fair during health emergencies | 0.751 | 0.712 |
|  | T3 | Belief that health decisions in Saudi Arabia are based on scientific evidence | 0.823 | 0.701 |
|  | T4 | Preference for official Saudi sources when social media information differs | 0.735 | 0.717 |
|  | T5 | Trust official national media more than social media for epidemic-related information | 0.645 | 0.728 |
|  | T6 | Willingness to follow public health instructions and encourage family to do the same | 0.744 | 0.714 |
|  | T7 | Belief that Saudi health authorities mainly act in the public’s health interests | 0.722 | 0.722 |
|  | T8 | Belief that the WHO mainly acts in the public’s health interests | 0.553 | 0.736 |
|  | T9_R | Caution and questioning motives behind some health recommendations (reverse-coded) | -0.322 | 0.853 |
|  | T10_R | Acceptability of not following policy if it clearly contradicts Islamic rules (reverse-coded) | -0.315 | 0.851 |
| Moral perceptions | M1 | Personal responsibility for protecting others during health emergencies | 0.464 | 0.670 |
|  | M2 | Taking recommended vaccines as a responsible action to protect the community | 0.563 | 0.648 |
|  | M3 | Acceptance of minor vaccine side effects to protect people at higher risk | 0.364 | 0.684 |
|  | M4 | Religious beliefs encourage following health guidance when supported by trusted religious authorities | 0.558 | 0.657 |
|  | M5 | Saudi cultural values support accepting health interventions that protect others | 0.558 | 0.657 |
|  | M6_R | Some preventive measures are more than what is really needed (reverse-coded) | 0.257 | 0.708 |
|  | M7_R | May not follow every health measure if the actual risk is very low (reverse-coded) | 0.209 | 0.715 |
|  | M8_R | Ignoring official health guidance can be morally acceptable in some situations (reverse-coded) | 0.125 | 0.738 |
|  | M9 | Spreading unverified health information during epidemics is morally unacceptable | 0.367 | 0.685 |
|  | M10 | People should follow health recommendations when linked to protecting others | 0.506 | 0.671 |
| Anticipated behavior | B1 | Willingness to follow preventive health measures and authority instructions in future epidemics | 0.699 | 0.869 |
|  | B2 | Maintaining hand hygiene and cleaning frequently touched surfaces in epidemic situations | 0.800 | 0.864 |
|  | B3 | Avoiding crowded places, unnecessary travel, and non-essential social visits during outbreaks | 0.832 | 0.861 |
|  | B4 | Practicing social distancing and wearing a face mask if recommended | 0.880 | 0.859 |
|  | B5 | Willingness to receive recommended vaccines in future epidemics | 0.515 | 0.885 |
|  | B6 | Ability and willingness to self-isolate if infected | 0.789 | 0.864 |
|  | B7 | Use of official Saudi digital health platforms during future epidemics | 0.844 | 0.861 |
|  | B8 | Reporting symptoms or testing promptly if infection is suspected | 0.807 | 0.862 |
|  | B9 | Avoiding sharing unofficial health information on social media during future outbreaks | 0.828 | 0.862 |
|  | B10_R | Future epidemics may not worsen even if some people do not fully follow guidance (reverse-coded) | -0.071 | 0.945 |

*Note. Reverse-coded items are identified by _R in the item label.*

**Appendix Table A10. Normality Tests for Main Scale Scores**

| **Scale** | **N** | **Kolmogorov-Smirnov statistic** | **p** | **Shapiro-Wilk statistic** | **p** |
| --- | --- | --- | --- | --- | --- |
| Public trust | 256 | 0.197 | < .001 | 0.844 | < .001 |
| Moral perceptions | 256 | 0.122 | < .001 | 0.965 | < .001 |
| Anticipated behavior | 256 | 0.238 | < .001 | 0.752 | < .001 |
| Overall total score | 256 | 0.164 | < .001 | 0.883 | < .001 |

**Appendix Table A11. Pearson Correlation Matrix of Age and Main Study Scores**

| **Variable** | **Age** | **Public trust** | **Moral perceptions** | **Anticipated behavior** | **Total score** |
| --- | --- | --- | --- | --- | --- |
| Age | 1 | 0.106 (.091) | 0.191 (.002) | 0.190 (.002) | 0.183 (.003) |
| Public trust |  | 1 | 0.677 (< .001) | 0.671 (< .001) | 0.872 (< .001) |
| Moral perceptions |  |  | 1 | 0.742 (< .001) | 0.898 (< .001) |
| Anticipated behavior |  |  |  | 1 | 0.909 (< .001) |
| Total score |  |  |  |  | 1 |

**Appendix Table A12. Spearman Rank Correlation Matrix of Age and Main Study Scores**

| **Variable** | **Age** | **Public trust** | **Moral perceptions** | **Anticipated behavior** | **Total score** |
| --- | --- | --- | --- | --- | --- |
| Age | 1 | 0.108 (.085) | 0.200 (.001) | 0.212 (< .001) | 0.189 (.002) |
| Public trust |  | 1 | 0.604 (< .001) | 0.611 (< .001) | 0.794 (< .001) |
| Moral perceptions |  |  | 1 | 0.764 (< .001) | 0.910 (< .001) |
| Anticipated behavior |  |  |  | 1 | 0.894 (< .001) |
| Total score |  |  |  |  | 1 |

**Appendix Table A13. Group Differences by Gender**

| **Outcome** | **Male Mean ± SD** | **Female Mean ± SD** | **Levene F** | **Levene p** | **t** | **df** | **p** |
| --- | --- | --- | --- | --- | --- | --- | --- |
| Public trust | 3.880 ± 0.692 | 3.938 ± 0.587 | 0.364 | .547 | -0.701 | 254 | .484 |
| Moral perceptions | 3.916 ± 0.630 | 3.994 ± 0.631 | 0.784 | .377 | -0.968 | 254 | .334 |
| Anticipated behavior | 4.288 ± 0.828 | 4.472 ± 0.531 | 3.490 | .063 | -1.982 | 254 | .049 |
| Overall total score | 4.028 ± 0.647 | 4.135 ± 0.515 | 0.807 | .370 | -1.396 | 254 | .164 |

**Appendix Table A14. One-Way ANOVA Summary for Demographic Differences in Study Scores**

| **Factor** | **Outcome** | **df** | **F** | **p** |
| --- | --- | --- | --- | --- |
| Marital status | Public trust | 3, 252 | 2.027 | .111 |
|  | Moral perceptions | 3, 252 | 4.348 | .005 |
|  | Anticipated behavior | 3, 252 | 7.845 | < .001 |
|  | Overall total score | 3, 252 | 5.434 | .001 |
| Education level | Public trust | 4, 251 | 2.011 | .093 |
|  | Moral perceptions | 4, 251 | 2.071 | .085 |
|  | Anticipated behavior | 4, 251 | 6.628 | < .001 |
|  | Overall total score | 4, 251 | 4.076 | .003 |
| Employment status | Public trust | 9, 246 | 1.554 | .130 |
|  | Moral perceptions | 9, 246 | 1.811 | .067 |
|  | Anticipated behavior | 9, 246 | 2.585 | .007 |
|  | Overall total score | 9, 246 | 2.267 | .019 |
| Household income | Public trust | 4, 203 | 1.259 | .287 |
|  | Moral perceptions | 4, 203 | 1.889 | .114 |
|  | Anticipated behavior | 4, 203 | 1.658 | .161 |
|  | Overall total score | 4, 203 | 1.922 | .108 |

**Appendix Table A15. Anticipated Behavior Level by Sociodemographic Characteristics**

| **Factor** | **Category** | **Total n** | **Low n (%)** | **Moderate n (%)** | **High n (%)** | **χ²** | **df** | **p** |
| --- | --- | --- | --- | --- | --- | --- | --- | --- |
| Gender | Male | 154 | 7 (4.5%) | 16 (10.4%) | 131 (85.1%) | 5.315 | 2 | .070 |
|  | Female | 102 | 1 (1.0%) | 5 (4.9%) | 96 (94.1%) |  |  |  |
| Marital status | Single / unmarried | 95 | 4 (4.2%) | 8 (8.4%) | 83 (87.4%) | 25.876 | 6 | < .001 |
|  | Married | 150 | 3 (2.0%) | 10 (6.7%) | 137 (91.3%) |  |  |  |
|  | Divorced | 6 | 0 (0.0%) | 0 (0.0%) | 6 (100.0%) |  |  |  |
|  | Widowed | 5 | 1 (20.0%) | 3 (60.0%) | 1 (20.0%) |  |  |  |
| Education level | Less than high school | 11 | 3 (27.3%) | 3 (27.3%) | 5 (45.5%) | 37.351 | 8 | < .001 |
|  | High school diploma | 41 | 2 (4.9%) | 1 (2.4%) | 38 (92.7%) |  |  |  |
|  | Bachelor's degree | 141 | 3 (2.1%) | 10 (7.1%) | 128 (90.8%) |  |  |  |
|  | Master's degree | 45 | 0 (0.0%) | 7 (15.6%) | 38 (84.4%) |  |  |  |
|  | Doctoral degree | 18 | 0 (0.0%) | 0 (0.0%) | 18 (100.0%) |  |  |  |
| Employment status | Government employee (civilian) | 46 | 1 (2.2%) | 4 (8.7%) | 41 (89.1%) | 35.773 | 18 | .008 |
|  | Healthcare worker | 55 | 0 (0.0%) | 2 (3.6%) | 53 (96.4%) |  |  |  |
|  | Education sector | 19 | 0 (0.0%) | 1 (5.3%) | 18 (94.7%) |  |  |  |
|  | Private sector employee | 32 | 0 (0.0%) | 2 (6.2%) | 30 (93.8%) |  |  |  |
|  | Self-employed / freelancer | 13 | 2 (15.4%) | 3 (23.1%) | 8 (61.5%) |  |  |  |
|  | Military / security sector | 16 | 2 (12.5%) | 0 (0.0%) | 14 (87.5%) |  |  |  |
|  | Housewife | 16 | 0 (0.0%) | 1 (6.2%) | 15 (93.8%) |  |  |  |
|  | Student | 32 | 0 (0.0%) | 6 (18.8%) | 26 (81.2%) |  |  |  |
|  | Retired | 13 | 2 (15.4%) | 1 (7.7%) | 10 (76.9%) |  |  |  |
|  | Unemployed / looking for work | 14 | 1 (7.1%) | 1 (7.1%) | 12 (85.7%) |  |  |  |
| Household income | Less than 3,000 SAR | 20 | 1 (5.0%) | 3 (15.0%) | 16 (80.0%) | 17.763 | 10 | .059 |
|  | 3,000-6,000 SAR | 22 | 3 (13.6%) | 1 (4.5%) | 18 (81.8%) |  |  |  |
|  | 6,001-10,000 SAR | 24 | 0 (0.0%) | 1 (4.2%) | 23 (95.8%) |  |  |  |
|  | 10,001-15,000 SAR | 49 | 1 (2.0%) | 5 (10.2%) | 43 (87.8%) |  |  |  |
|  | More than 15,000 SAR | 93 | 1 (1.1%) | 4 (4.3%) | 88 (94.6%) |  |  |  |
|  | Prefer not to answer | 48 | 2 (4.2%) | 7 (14.6%) | 39 (81.2%) |  |  |  |

*Note. Percentages are calculated within row categories.*

**Appendix Table A16. Anticipated Behavior Level by Public Trust and Moral Perception Levels**

| **Factor** | **Level** | **Total n** | **Low n (%)** | **Moderate n (%)** | **High n (%)** | **χ²** | **df** | **p** |
| --- | --- | --- | --- | --- | --- | --- | --- | --- |
| Public trust level | Low | 12 | 4 (33.3%) | 5 (41.7%) | 3 (25.0%) | 68.280 | 4 | < .001 |
|  | Moderate | 51 | 2 (3.9%) | 8 (15.7%) | 41 (80.4%) |  |  |  |
|  | High | 193 | 2 (1.0%) | 8 (4.1%) | 183 (94.8%) |  |  |  |
| Moral perceptions level | Low | 5 | 4 (80.0%) | 1 (20.0%) | 0 (0.0%) | 147.222 | 4 | < .001 |
|  | Moderate | 56 | 3 (5.4%) | 16 (28.6%) | 37 (66.1%) |  |  |  |
|  | High | 195 | 1 (0.5%) | 4 (2.1%) | 190 (97.4%) |  |  |  |

*Note. Percentages are calculated within row categories.*

**Appendix Table A17. Multiple Linear Regression Predicting Anticipated Behavior**

| **Block** | **Predictor / Statistic** | **B / value** | **SE** | **Standardized β** | **t** | **95% CI for B** | **p** |
| --- | --- | --- | --- | --- | --- | --- | --- |
| Model summary | R | 0.777 |  |  |  |  |  |
|  | R² | 0.604 |  |  |  |  |  |
|  | Adjusted R² | 0.601 |  |  |  |  |  |
|  | F-test | F(2, 253) = 192.693 |  |  |  |  | < .001 |
| Coefficients | Constant | 0.578 | 0.195 |  | 2.962 | 0.194, 0.963 | .003 |
| Coefficients | Public trust | 0.348 | 0.060 | 0.311 | 5.784 | 0.229, 0.466 | < .001 |
| Coefficients | Moral perceptions | 0.614 | 0.062 | 0.532 | 9.887 | 0.492, 0.737 | < .001 |

**Appendix Table A18. Adjusted General Linear Model Predicting Anticipated Behavior**

| **Effect** | **df** | **Type III SS** | **F** | **p** | **Partial η²** |
| --- | --- | --- | --- | --- | --- |
| Model summary | N = 208 | R² = 0.671 | Adjusted R² = 0.628 |  |  |
| Age | 1, 183 | 1.248 | 6.594 | .011 | 0.035 |
| Public trust | 1, 183 | 5.230 | 27.627 | < .001 | 0.131 |
| Moral perceptions | 1, 183 | 7.588 | 40.082 | < .001 | 0.180 |
| Gender | 1, 183 | 0.960 | 5.071 | .026 | 0.027 |
| Marital status | 3, 183 | 2.485 | 4.376 | .005 | 0.067 |
| Education level | 4, 183 | 4.761 | 6.288 | < .001 | 0.121 |
| Employment status | 9, 183 | 1.173 | 0.689 | .719 | 0.033 |
| Household income | 4, 183 | 0.930 | 1.228 | .300 | 0.026 |

*Note. Participants selecting “Prefer not to answer” for household monthly income were excluded from this adjusted model because this value was treated as missing in SPSS.*

## Supplementary Figures

**Appendix Figure A1. Response profiles across anticipated behavior items.**

**Appendix Figure A2. Strongly agree responses across positive anticipated behavior items.**

**Appendix Figure A3. Histogram of the public trust mean score.**

**Appendix Figure A4. Histogram of the moral perceptions mean score.**

**Appendix Figure A5. Histogram of the anticipated behavior mean score.**

**Appendix Figure A6. Anticipated behavior by significant sociodemographic groupings.**

**Appendix Figure A7. Focused response distribution for willingness to receive recommended vaccines in future epidemics.**
